# Clinical and biological significance of circulating tumor cells, circulating tumor DNA, and exosomes as biomarkers in colorectal cancer

**DOI:** 10.18632/oncotarget.17184

**Published:** 2017-04-18

**Authors:** Shiyu Jia, Rui Zhang, Ziyang Li, Jinming Li

**Affiliations:** ^1^ Peking University Fifth School of Clinical Medicine, Beijing, People's Republic of China; ^2^ National Center for Clinical Laboratories, Beijing Hospital, National Center of Gerontology, Beijing, People's Republic of China; ^3^ Beijing Engineering Research Center of Laboratory Medicine, Beijing Hospital, Beijing, People's Republic of China; ^4^ Graduate School, Peking Union Medical College, Chinese Academy of Medical Sciences, Beijing, People's Republic of China

**Keywords:** colorectal cancer (CRC), liquid biopsy, circulating tumor cells (CTCs), circulating tumor DNA (ctDNA), exosomes

## Abstract

Colorectal cancer (CRC) has been the fourth leading cause of cancer-related mortality worldwide. Owing to clonal evolution and selection, CRC treatment needs multimodal therapeutic approaches and due monitoring of tumor progression and therapeutic efficacy. Liquid biopsy, involving the use of circulating tumor cells (CTCs), circulating tumor DNA (ctDNA), and exosomes, may offer a promising noninvasive alternative for diagnosis and for real-time monitoring of tumor evolution and therapeutic response compared to traditional tissue biopsy. Monitoring of the disease processes can enable clinicians to readily adopt a strategy based on optimal therapeutic decision-making. This article provides an overview of the significant advances and the current clinical and biological significance of CTCs, ctDNA, and exosomes in CRC, as well as a comparison of the main merits and demerits of these three components. The hurdles that need to be resolved and potential directions to be followed with respect to liquid biopsies for detection and therapy of CRC are also discussed.

## INTRODUCTION

With a global incidence of about 1.4 million individuals and 693,900 deaths reported in 2012, colorectal cancer (CRC) is the third most common type of cancer and the fourth most common cause of cancer deaths worldwide [1, 2]. The conventional treatment strategies for CRC include surgery, neoadjuvant radiotherapy (rectal cancer patients), and adjuvant chemotherapy (stage III/IV and high-risk stage II colon cancer patients) [3]. Unfortunately, about 50% of the patients with CRC are diagnosed only at late stages, thereby significantly reducing the availability of different treatment options. Despite the strong inheritance factor related to it, CRC is commonly sporadic and proceeds slowly over 10 years, hence correct diagnosis of the disease-stage becomes crucial for prognosis. The 5-year relative survival ranges from greater than 90% in patients with early-stage localized disease (Stages I and II) to only slightly greater than 13% in patients with late-stage CRC [4]. Nowadays, targeted therapy is one of the principal modes of cancer treatment, which has had varying degrees of success owing to the diverse range of resistance mechanisms [5, 6]. Within a few months into treatment, it is common to see changes in the tumor and development of therapeutic resistance [7].

Therefore, screening and early detection of CRC remain clinical dilemmas and are critically important for raising the chances of a more suitable treatment leading to better long-term survival. Several approaches involving colonoscopy, sigmoidoscopy, fecal occult blood test (FOBT) [2, 8], serum biomarkers such as carcinoembryonic antigen (CEA) and carbohydrate antigen 19-9 (CA19-9) [9], together with Magnetic Resonance Imaging (MRI) and Computed Tomography (CT) [10] are commonly used to screen or diagnose CRC. However, the use of CEA or CA19-9 is limited by its low sensitivity and specificity [11]. Because of the inherent characteristics of CT or MRI, some early tumor dissemination or micro-metastases may be missed in tumor detection, so is the case with colonoscopy with a higher risk of complications. Although colonoscopy is still the most effective method to diagnose CRC, this approach has poor patient compliance and might not provide real-time monitoring of tumor progression and therapeutic response.

**Figure 1:**
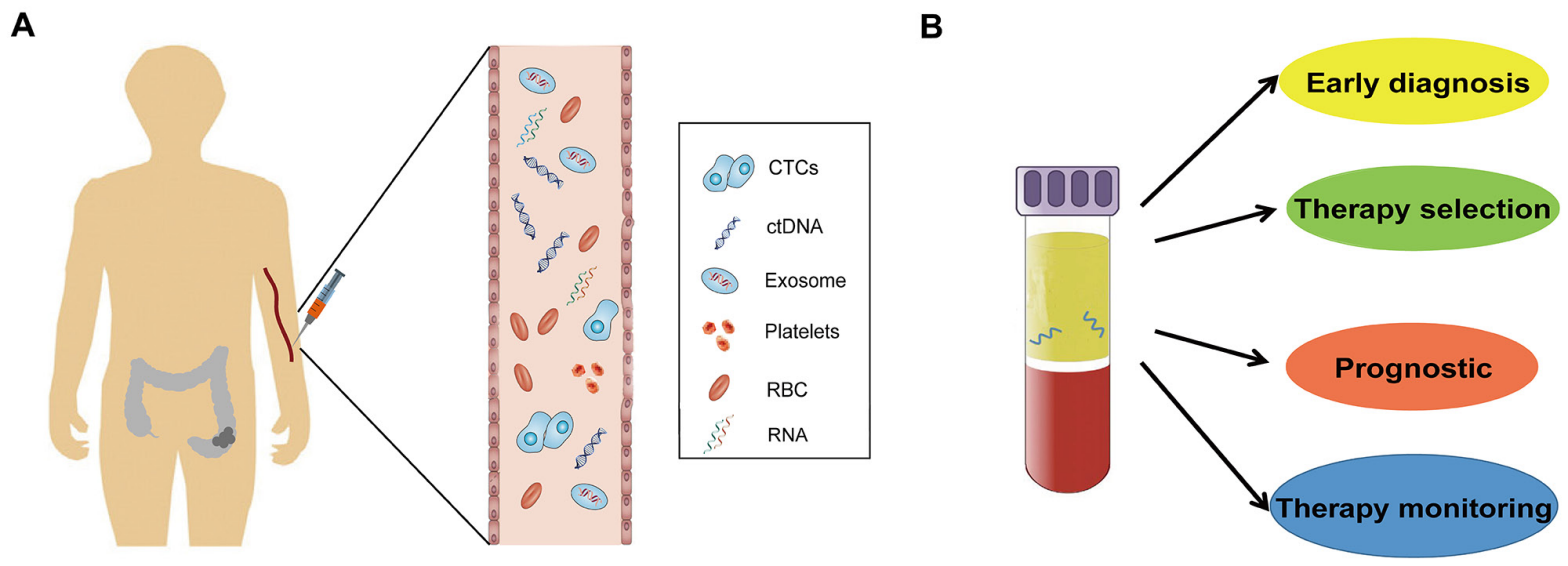
Circulating biomarkers in CRC patient **(A)** and the clinical application of liquid biopsy **(B)**.

Therefore, it is necessary to identify ideal bio-markers that can be used for the early diagnosis, detection of recurrence, and monitoring of metastasis for CRC. Using body fluid samples, liquid biopsies may be the ideal approach for the detection of CTCs or for the products of primary or metastatic tumors. The advent of liquid biopsy as an alternate, easy, quick, convenient, and minimally invasive method has shown tremendous potential to help clinicians achieve early diagnosis of cancer and guide decision-making during the course of treatment. It is expected to be an informative or easily accessible tool to provide comprehensive information of tumors beyond the invasive tissue biopsies. The main approaches to liquid biopsies include the detection of CTCs [12], analysis of ctDNA or RNA [13, 14], and the capture of exosomes that are secreted by tumors [15] (Figure 1).

This review summarizes and discusses the current clinical and biological significance of CTCs, ctDNA, and exosomes in CRC. Additionally, the review covers significant advances and limitations of liquid biopsy in the clinical applications, discusses the hurdles that need to be resolved, and provides potential directions for the detection and therapy of CRC.

## ORIGINS, MERITS, AND DEMERITS OF CTCS/ CTDNA/ EXOSOMES

Over the past few decades, many procedures have been developed for liquid biopsy ranging from the use of CTCs and ctDNA or RNA to that of exosomes and platelets [16, 17] detection, requiring time to judge and improve their effects. These biomarkers have several advantages over traditional tissue biopsy and their shortcomings, if any, need to solve (Table 1). With a large number of methods available for the detection of circulating biomarkers, there has not yet been a consensus on both the ideal technical method and clinical application.

**Table 1:** Merits and demerits of CTCs /ctDNA/exosomes

	Merits	Demerits
CTCs	Identified morphologically and molecular characterization	Low-input amounts and isolate rare cells with limited capture techniques
	Allows immuno-labeling based approaches	Methodological limitations(sensitivity and specificity) and standardization
	Allow functional *in vitro*/*in vivo* assays	Heterogeneity of the CTC populations
ctDNA	Quick-renewed	Discrimination of ctDNA from normal cfDNA
	Short half-life	Extremely low levels of ctDNA
	More sensitive for detection of tumor status	No functional assays
	Identified molecular characterization	Methodological limitations(sensitivity and specificity) and standardization
Exosomes	Inherent stability, maintain the integrity of contents	Isolation and purification of exosomes
	With concentrations of ≥10^9^ vesicles/mL	The enrichment of specific markers within the exosomes
	Abundant contents	Methodological limitations(sensitivity and specificity) and standardization
	As vectors for anti-tumor therapy of gene or drug delivery	

### Circulating tumor cells

CTCs are circulating tumor cells that are shed into the bloodstream from tumors and have the potential to cause metastatic lesions [18, 19]. The CTC pool in cancer patients may include not only epithelial tumor cells, but also epithelial–mesenchymal transition (EMT) tumor cells, hybrid (epithelial/EMT+) tumor cells, irreversible EMT+ tumor cells, and circulating tumor stem cells (CTSCs) [20]. They can be single or clustered together in circulation, or form metastases. CTCs can be separated from normal blood cells by physico-chemical characteristics or cell surface molecules. As real-time and noninvasive surrogates, the isolation and detection of CTCs is one of the most promising methods to help patients with CRC obtain early diagnosis and accurately predict metastasis or recurrence of malignancies. The most widely used CTC enumeration platform, CellSearch™ System (Veridex LLC, NJ, USA), has been approved for clinical use by the Food and Drug Administration (FDA) in the United States to monitor patients with metastatic colorectal, prostate, and breast cancers [21–25]. In order to study the functional attributes of CTCs, Aceto et al. captured and cultured the CTCs and implanted them into mouse and proved that rare CTC clusters, shed from tumor *in situ*, contributed to the metastasis through plakoglobin-dependent intercellular adhesion [26].

Although the underlying advantages of CTCs make it a promising tool to help monitor the dynamic course of disease, there are some potential limitations in the existing detection techniques [27–29] and from the heterogeneity of the CTCs. Methods that rely on the physical properties of CTCs for their capture, typically achieve high capture efficiency which is greater than 80%; however, not all CTCs are larger than nucleated blood cells, for example, the tumor cells undergoing apoptosis or those in EMT may be smaller in size [29]. Methods that capture positive biomarkers of CTCs are flawed too, causing the actual CTC load to be underestimated and CTC populations that are highly relevant to the disease progression to be missed [30]. Epithelial cell adhesion molecule (EpCAM) expression was approximately 10-fold lower on CTCs compared to on primary and metastatic tissues, suggesting that it is dependent on the local microenvironment and is down-regulated on CTCs [31]. This may be the reason why CTCs were undetected by the CellSearch™ System in a significant proportion of patients with CRC [32]. Although the CellSearch™ is the only FDA-approved technology applied for CTC enrichment, it cannot capture non-epithelial CTCs, such as the CTCs that have undergone EMT, resulting in a missed detection. Moreover, RNA can be extracted from CTCs and detected for guiding treatment decision. The real-time reverse transcription polymerase chain reaction (RT-PCR) assay targeting mRNA may have the advantage of improved sensitivity, compared to cell-based assays [33, 34]. Until date, no ideal surface marker from CTCs covers all the stages or types of cancer.

### Circulating tumor DNA

Circulating free DNA (cfDNA) is a natural phenomenon and is thought to originate from the apoptosis and necrosis of normal and tumor cells, and secretion of tumor cells has also been suggested as a potential source [28, 35]. The fragments of ctDNA showed an enrichment of 166 bp, clearly corresponding to the size of DNA wrapped around the nucleosomes [36, 37]. The mean half-life for circulating fetal DNA has been found to be 16.3 min (range 4–30 min) and they are eliminated within 2 hours [38]. Similarly, ctDNA also has a short half-life and the rapid turnover time allow clinicians to monitor the dynamic changes in tumor within a matter of only few hours rather than weeks [39]. Therefore, ctDNA is a prospective material in cancer research with vast amounts of information. Traditional detection methods such as Sanger sequencing and quantitative PCR can only test higher concentrations of ctDNA due to its low sensitivity. The emergence of BEAMing (beads, emulsion, amplification and magnetics) and digital PCR (dPCR) allows researchers to detect as low as 0.01% ctDNA with known anomalous sequence in circulation [6]. Fortunately, the next generation sequencing (NGS) technology addresses this issue, which can monitor all possible cancer mutations and identify rare mutation variants. Additionally, benign tumors and nonneoplastic conditions do not generally give rise to ctDNA, and therefore may not influence the detection [40].

Many studies have demonstrated that the level of cfDNA is substantially higher in patients with cancer than that in healthy individuals or patients with benign diseases, and it seems to increase with tumor stage [41, 42]. ctDNA analysis of Epstein–Barr virus (EBV) has been certified for the early screening and detection of nasopharyngeal carcinoma (NPC) in patients without any former indications [43, 44]. Although quantification of ctDNA may not be informative enough for tumor diagnosis by itself, a decrease or increase in its level after therapy may be a prognostic factor for residual disease and recurrence [43]. The first prospective study using colonoscopies as the reference standard to assess the accuracy of circulating methylated *SEPT9* DNA (mSEPT9) for detecting CRC in a screening population was conducted by Church et al. [45]. Results from 53 CRC cases and 1457 subjects without CRC yielded a standardized sensitivity of 48.2% and specificity of 91.5% [45]. Although early detection and screening strategies based on ctDNA are promising, it can only provide limited information for cancer owing to the related technical challenges and other obstacles [46]. Therefore, the use of ctDNA detection as a CRC screening tool requires further exploration. Nevertheless, the detection of somatic mutations in ctDNA can offer the potential for better diagnostic accuracy and guiding treatment decisions [47].

### Exosomes

Exosomes, which are well known for intercellular communication, are small membrane vesicles (30–100 nm) released from diverse cell types under both normal and pathological conditions, that can horizontally transfer functional biomolecules (i.e., DNA, RNA, proteins, and lipids) to recipient cells [48–50]. The exosomal membrane reflects the cell plasma membrane in several ways and can therefore be immuno-isolated utilizing significantly enriched proteins on the membrane surface, e.g., A33 affinity-isolation of CRC cell line exosomes [49]. It may not only serve as an important regulatory mechanism during cancer development and progression, such as promoting adhesion, triggering signaling pathways and inflammatory responses, or immune escape, but also play significant role in the diagnosis, treatment assessment, and prognosis of tumor. Besides, exosomes can be directly used as vectors for cancer intervention through gene or drug delivery.

Previous studies by Peinado and colleagues have demonstrated that exosomes played vital roles in vascular leakiness, inflammation, and bone-marrow progenitor cell recruitment during pre-metastatic niche formation and metastasis itself [51]. Subsequently, data have indicated that integrin expression profiles of plasma exosomes, isolated from cancer patients, could be used as prognostic factors to predict sites of future metastasis [52]. Indeed, exosomes perform a variety of extracellular functions that involve interactions with the cellular microenvironment, such as immunological mediation, cell recruitment, and horizontal transfer of genetic material [53]. Interestingly, with concentrations as high as or above 10^9^ vesicles/mL in blood, the number of exosomes secreted by tumor cells correlates to their malignant behavior [50, 53]. Therefore, to examine whether exosomal DNA (exoDNA) could be utilized as a surrogate for tumor tissues or cells in the detection of tumor-specific genetic mutations just as it is with ctDNA, researchers have tested the exoDNA isolated from various cancer cell lines to detect the *BRAF* (V600E) mutation and anti-epidermal growth factor receptor (*EGFR*) mutation, using allele-specific polymerase chain reaction (AS-PCR). The findings demonstrated that exoDNA could reflect the mutational status of the parental cell lines [54]. However, some confounding factors, such as HIV-1 particles, may decrease the accuracy and reliability of the detected results in the analysis of exosomes. Evidently, a relationship between exosomes and tumor is like that of a seed and its plant, and therefore, exosomes can be expected to serve as useful biomarkers owing to their remarkable stability in fluids.

## CIRCULATING TUMOR CELLS, CIRCULATING TUMOR DNA, AND EXOSOMES AS BIOMARKERS IN COLORECTAL CANCER

Having discussed the advantages and disadvantages of CTCs, ctDNA, and exosomes in different aspects, it is clear that these hold promise for researchers attempting to monitor tumor-specific changes during the course of cancer. This may also help clinicians to carry out noninvasive real-time assessment in various clinical settings, including diagnosis, therapy monitoring, and prognosis (Table 2).

**Table 2:** Application value of CTCs/ctDNA/exosomes in CRC

Material	Biomarkers	Potential clinical utility	Methods	References
CTCs	CK-19, EpCAM	Prognostic	CK19-Epispot and CellSearch™	[58]
	EpCAM	Predictive and prognostic	CellSearch™	[22, 23][59]
	CEA, CK19, and CK20	Prognostic	RT-PCR	[33]
	CEA/CK/CD133	Prognostic	RT-PCR	[34]
	EpCAM, CKs; VIM, TWIST1, AKT2, SNAI1	Prognostic	CanPatrol^TM^	[60]
	CD133, EpCAM, CD26, CD44v6	Prognostic(functional research)	Drug sensitivity analysis of CTC lines	[62]
	*KRAS*, *BRAF*, and *PI3KCA*	Prognostic	Label-free Vortex technology	[28]
	EpCAM	Screening high-risk stage II CRC patients	CellSearch™	[66]
ctDNA	Methylated *SEPT9*	Screening and early detection	Duplicate real-time PCRs	[45]
	*APC*, *KRAS*, *TP53*, and *PI3KCA*	Prognostic and therapy monitoring	BEAMing, real-time PCR	[39]
	A modest panel of 15 genes	Predictive	Safe-SeqS	[67]
	*APC*, *KRAS*, *TP53*	Predictive	Safe-SeqS	[68]
	Methylated *WIF1* and *NPY*	Prognostic	dPCR	[69]
	Methylated *ALU83*, *ALU244*, *OSMR*, and *SFRP1*	Diagnostic and prognostic	ALU-based real-time PCR, methylation-specific real-time PCR	[70]
	*KRAS*	Therapy selection and monitoring	BEAMing, real-time PCR, and NGSeq,	[71]
	*KRAS*	Therapy selection and monitoring	BEAMing and real-time PCR	[7]
	*KRAS, BRAF*	Therapy selection and monitoring	Real-time PCR	[72]
	*KRAS*, *NRAS*, *MET*, *ERBB2*, *FLT3*, *EGFR*, and *MAP2K1*	Therapy selection and monitoring	dPCR and NGS	[74]
	*KRAS*, *BRAF*	Therapy selection and monitoring	ARMS-qPCR	[75]
Exosomes	miRNAs	Diagnostic	miRNA microarray analysis and qRT-PCR	[79]
	miRNAs	Prognostic	Microarray and CGH analysis and qRT-PCR	[80]
	Proteins	Diagnostic	Mass spectrometry and western blotting	[81]
	Proteins	Prognostic (function research)	Proteomics measurements and western blotting	[82]
	Microvesicles (Fas ligand and TNF)	Prognostic and therapeutic(function research)	Flow cytometry, western blotting, and immunoelectron microscopy	[89]

### Clinical applications of CTCs in colorectal cancer

A milestone in CTC research (CellSearch™) in patients with metastatic breast cancer (MBC) was reported in The New England Journal of Medicine in 2004 [55]. The researchers discovered that five or more CTCs/7.5 mL were detected in 49% (87/177) of the patients. Another study on patients with MBC treated with standard therapy found that 46.9% (911/1944) of the patients had a CTC count of ≥5 CTCs/7.5 mL at baseline [56]. These patients with decreased progression-free survival (PFS) and overall survival (OS) were compared with patients with less than five CTCs per 7.5 mL at baseline. These data confirmed the number of CTCs before treatment is an independent predictor of PFS and OS in MBC patients. However, the level of CTCs was lower in peripheral blood of colon cancer patients compared to that in breast or prostate cancer patients, making it more difficult to detect CTCs in colon cancer [57].

Deneve et al. demonstrated that the CTC counts were significantly higher in mesenteric blood than in the peripheral blood, which meant that the liver acts as a filter for CTCs, and the follow-up analysis showed that localized colon-cancer patients with high CTC counts have an unfavorable outcome (n =60) [58]. By contrast, fewer CTCs meant a longer median PFS and OS. Therefore, collection of mesenteric blood may improve the detection ratio of CTCs. Similar observations were made in other cancer types, including prostate and breast cancers [55, 59]. In a published study Bork et al. showed for the first time that preoperative CTC detection by the standardized CellSearch™ System is a strong and independent prognostic factor for disease progression and survival in patients with non-metastatic CRC, although results show a significantly lower detection rate of CTC in patients with non-metastatic CRC [60]. Another prospective, multicenter study evaluated CTCs in 430 patients with metastatic CRC (mCRC) at baseline and after first-, second-, or third-line therapy by using a CellSearch™ System [22]. Patients with unfavorable (≥3 CTCs/7.5 mL) compared with favorable (<3 CTCs/7.5 mL) baseline CTCs had shorter median PFS and OS. They demonstrate that CTCs can serve as both prognostic and predictive factors for patients with mCRC, and that the baseline levels of three or more CTCs /7.5 mL and follow-up CTC levels are strong independent prognostic factors for inferior PFS and OS [22]. A meta-analysis suggested that for patients with CRC, CTC mRNA detection, targeting CEA, cytokeratin (CK) 19, and CK20, using RT-PCR could serve as a prognostic indicator and a mode of CRC staging [33]. Crucially, a study demonstrated that the detection of CEA/CK/CD133 mRNA in the circulating blood of patients with Dukes’ stage B and C CRC who require adjuvant chemotherapy is a useful tool for determining which patients are at high risk for recurrence and poor prognosis [34]. Thus, CTC could serve as a much earlier prognostic and predictive factor than standard anatomical or functional imaging studies to monitor tumor burden in real-time.

Recently, a large cohort of 1203 patients was evaluated for the expression of EMT markers in CTCs by using CanPatrol^TM^ CTC technique. Biophenotypic (epithelial/mesenchymal) CTCs as well as mesenchymal CTCs positively correlated with both clinical stage and lymph node metastasis and distant metastasis, suggesting that CTCs displaying a mesenchymal phenotype would denote a more aggressive and metastatic potential [61]. Their findings suggest that the combination of epithelial (EpCAM, CKs) and mesenchymal (VIM, TWIST1, AKT2, and SNAI1) markers in CTCs analysis may offer valuable aid for tumor staging and metastasis evaluation in patients with CRC, which may be superior to CellSearch™ System [61].

Cancer stem cells are capable of seeding, recirculation, and evolution in metastatic clones, which causes clinical progression of the disease [19]. The first permanent CRC cell line, named CTC-MCC-41 and obtained from the CTCs of a patient with colon cancer, was established by Laure et al. in 2015 [62]. Genome, transcriptome, proteome, and secretome level analyses and functional studies showed that CTC-MCC-41 cells derived from the bone marrow based on osteoprotegerin expression, showed an intermediate epithelial/mesenchymal phenotype with stem cell–like characteristics and could induce *in vitro* angiogenesis and tumors in immune-deficient mice [62]. By establishing CTC lines from the blood of patients with mCRC, another study also demonstrated that patient-derived colorectal CTCs own all the functional attributes of CTSCs [63]. The cytotoxicity assay confirmed the potential application of this model to predict individual patient-drug response. Strikingly, the CTCs cultured model was simple and took less than a month from blood collection to drug testing [63]. Therefore, the detection, *in vitro* culture, and molecular characterization of CTCs should divulge the prognosis of mCRC as well as monitor the drug response, in addition to early detection of disease progression with new metastasis.

National Comprehensive Cancer Network (NCCN) highly recommends that patients with mCRC should be tested for the *RAS* or *BRAF* mutation status in primary or metastatic tumors. The mutation status of *KRAS*, *BRAF*, and *PI3KCA* could affect the treatment response to *EGFR* monoclonal antibodies or small molecule inhibitors and have treatment-independent prognostic value [28, 64, 65]. Fabbri et al. demonstrated, for the first time, the feasibility of analyzing pure CTCs at the molecular level and avoiding lymphocyte contamination using a DEPArray, a dielectrophoresis-based platform, as well as a *KRAS* discordance between CTCs and primary tissue cancer after 100% pure cell recovery and sequencing [66]. The unexpected CTC-primary tissue discordance may be due to the presence of intratumor heterogeneity and multiple metastatic clones, which are disseminated very early during disease progression and remain dormant for years [66]. In a recent study including 23 matched CTC and ctDNA samples, *KRAS*, *BRAF*, and *PIK3CA* hotspot mutations were analyzed by a label-free platform. Researchers found a concordance of 78.2% for *KRAS*, 73.9% for *BRAF*, and 91.3% for *PIK3CA* mutations [28]. In general, complementary assessment of both CTCs and ctDNA should be more superior to assess dynamic tumor profiles.

Thus, in primary or non-metastatic CRC, the existence of CTCs might indicate poor prognosis; while in advanced or metastatic CRC, there should be a positive correlation between the level of CTCs with the tumor progression and poor outcomes; CTCs can guide treatment decisions and assess treatment responses during the course of therapy; the molecular analysis of CTCs may predict drug resistance and the selection of anticancer drugs [61]. Gazzaniga et al. evaluated the CTCs in 37 high-risk patients with stages II-III CRC after primary tumor resection and before the start of adjuvant therapy. Results showed 87.5% (7/8) CTC-positive patients had N1–2 disease and stage III CRC, whereas only one patient who experienced the progression of disease had a high risk stage II disease [67]. The data suggest that the detection of CTCs might help screen high-risk stage II CRC candidates for adjuvant chemotherapy, after enumerating CTCs with the CellSearch system [67]. Whether CTCs detection can differentiate patients with N+ disease and some of the patients with N+ disease can skip chemotherapy need to be further explored. Although the detection of CTCs as a biomarker of tumor has been well accepted, strategies involving the use of CTCs to completely guide the treatment decisions are in progress. Remarkably, the sensitivity and specificity of the CTC detection methods could pose as an obstacle that needs to be urgently resolved.

### Clinical applications of ctDNA in colorectal cancer

Diehl et al. applied BEAMing to quantify ctDNA in CRC patients undergoing multimodality therapy. Compared to CEA, the levels of ctDNA were more impressive in the prediction of recurrence (P=0.03), which certified that fluctuations of ctDNA could be used to monitor the course of therapy in patients with mCRC undergoing surgery or chemotherapy [39]. In another study involving 53 patients with mCRC receiving standard first-line chemotherapy, the concordance between ctDNA (Safe-SeqS) and tumor tissue was found to be 92.3%. In general, ctDNA appeared to be an early biomarker to infer the tumor burden of patients with CRC during first-line chemotherapy and predicts an earlier therapeutic reaction than radiographic approaches [68]. In addition, a prospective cohort study of 230 patients with resected stage II colon cancer demonstrated that the detection of ctDNA after resection provides direct evidence of the residual disease and identifies patients at very high risk of recurrence, thus being superior to other clinico-pathological measures [69]. These findings establish the rationale for measuring ctDNA for using as a monitoring tool for recurrence and guiding clinical decisions.

Until date, several studies have focused on the detection of methylated DNA in patients with CRC. Garrigou and colleagues reported that hypermethylation of *WIF1* (WNT inhibitory factor 1) and *NPY* (neuropeptide Y) was significantly higher in the tumor tissue compared to that in normal tissue and these methylated ctDNA (Met ctDNA) were detectable throughout the tumor progression, with their fraction being correlated to the tumor stage [70]. Therefore, MetctDNA could be a promising surrogate marker for tumor follow-up in patients with CRC, which means that *WIF1* and *NPY* could instead be tumor-specific mutations. Recently, Bedin et al. found that the adenoma and methylated cfDNA in patients with CRC, showed a higher quantity of ALU83 and ALU244 [71]. In this study, *OSMR* and *SFRP1* methylation of cfDNA was also significantly higher in advanced CRC compared to that in the adenoma and control samples. This combination can improve the diagnostic efficiency and prognosis for CRC.

Misale et al. demonstrated, for the first time, that *KRAS* mutations are frequent drivers of acquired resistance to cetuximab in CRC, and indicated that the emergence of *KRAS* mutant clones can be detected, non-invasively, months prior to radiographic progression [72]. They also suggested early initiation of an MEK inhibitor as a rational strategy for delaying or reversing drug resistance [72]. The same result was reported by Diaz et al. [7]. They also found that each relatively large metastatic lesion was expected to contain a subclone comprising hundreds or thousands of cells with one of ˜42 mutations conferring resistance to the antibody, making resistance a fait accompli; the time to recurrence is simply the interval required for the subclone to repopulate the lesion [7]. The first blinded prospective multicenter study compared the mutation status of *KRAS* and *BRAF* in CRC tumor tissue, using routine gold-standard methods, and in ctDNA, using a quantitative PCR-based method. cfDNA analysis showed 100% specificity and sensitivity for the *BRAF V600E* mutation and 98% specificity and 92% sensitivity with a concordance value of 96% for the *KRAS* mutation, compared with a tumor-tissue analysis [73]. To conclude, the cfDNA analysis may advantageously replace tumor-section analysis and expand the scope of the management of personalized cancer care [73].

As a result of clonal evolution and selection [74], the ctDNA profiles of CRC patients who benefit from multiple challenging with anti-*EGFR* antibodies, exhibit pulsatile levels of mutant *KRAS*. Results revealed that the CRC genome adapts dynamically intermittent drug schedules and provided a molecular explanation for the efficacy of rechallenge therapies based on *EGFR* blockade [75]. Because of the clinical significance of the *KRAS* gene, *KRAS* wild type status in the primary tumor is a prerequisite for treatment modality of patients with mCRC with targeted therapy apart from the best supportive care alone [64, 73]. The previous study including 108 patients with mCRC monitored the number of mutant *KRAS* or *BRAF* alleles in the plasma at baseline and before each cycle of the third-line treatment with cetuximab and irinotecan. cfDNA and *KRAS* levels was found to decrease from baseline to cycle 3 and increased in progress (P = 0.08), while the loss of mutations was associated to the benefit of treatment, whereas the appearance of mutations during therapy may be correlated to acquired resistance in primary wild-type disease [76]. The appearance of *KRAS* mutation in wild-type tumors indicated a shift for the poor progression, while some patients with CRC had tumors that contained mutation but wild-type *KRAS* in plasma proved to have responded better to the therapy [76].

Most published studies of ctDNA have evaluated a single type of tumor mutation; however, numerous cancers have been reported with several undefined mutations. Further, the detection of a known anomalous gene could not completely reflect the heterogeneity of the tumor or replace the gold standard for tissue biopsy. A study explored the feasibility of Ion Torrent PGM (IonPGM) targeted NGS on cfDNA of mCRC patients undergoing colorectal liver metastasectomy [6]. The panel covered 21 most prevalent and relevant genes in CRC, known until date. Results showed that using NGS with IonPGM to detect cfDNA is feasible; however, it had limited sensitivity in the detection of all somatic mutations present in the tumor, especially in the case of unknown mutations. More importantly, true somatic mutations were present in normal-appearing tumor adjacent tissue, which implies that occult and potential lesions lead to recurrence. A study concluded that exome-wide analysis of ctDNA could complement the current invasive biopsy method to identify mutations related to acquired drug-resistance in advanced cancers [77].

Although increasing the sequencing depth of NGS could make the results more accurate, such an approach will be more time-consuming and costly, which is an obstacle for practical clinical application. However, whole genome sequencing with an appropriate depth of cfDNA could be available for clinical purposes, which can remedy the restrictions of dPCR. Because of the 0.01% error rate of DNA polymerases in the process of amplification, PCR or NGS-based methods that depend on amplification are limited. The newer sequencing technologies such as nanopore sequencing do not require nucleotides, polymerases, or ligases and have the potential of generating very long read-lengths (>10,000–50,000 nt), which might be a more competitive and portable technology for clinical applications [78, 79].

### Clinical applications of exosomes in colorectal cancer

Lately, exosomes have greatly attracted researchers’ attention as potential biomarkers of cancer. A study, performed at Kanazawa University on microarray-based profiling of exosomal microRNAs (miRNAs) in sera from patients with primary CRC, healthy controls and cancer cell lines, validated CRC-associated exosomal miRNAs in an independent sample set using quantitative real-time RT-PCR [80]. They found that the serum exosomal levels of seven miRNAs (let-7a, miR-1229, miR-1246, miR-150, miR-21, miR-223, and miR-23a) were significantly higher in patients with primary CRC even in the early stage than that in healthy controls, which was found to decrease significantly after surgical resection. Colon-cancer cell lines secreted significantly higher levels of miRNAs than the normal colon-derived cell line *in vitro* [80]. To summarize, specific exosomal miRNA can reflect pathological changes in CRC and are therefore promising biomarkers for non-invasive diagnosis of CRC. Similarly, in order to identify specific miRNAs in exosomes as a prognostic factor of recurrence in CRC, Matsumura and colleagues explored miRNA expression profiles and copy number aberrations with microarray and array comparative genomic hybridization (CGH) analysis to confirm exosomal miRNAs in two serum sample sets by qRT-PCR [81]. The study found that the level of exosomal miR-17-92a cluster in serum was associated with the recurrence of CRC. Exosomal miR-19a was found to have significantly increased in patients with CRC compared to in healthy individuals, with gene amplification, and the patients with CRC with high exosomal miR-19a expression showed poorer prognoses than the low expression group (P < .001), which might be a promising prognostic biomarker for recurrence in patients with CRC [81].

Recently, Yanyu Chen et al. presented a quantitative proteomics analysis of purified exosomes from the serum of patients with CRC and normal volunteers, and identified 918 proteins with an overlap of 725 Gene IDs in the Exocarta proteins list [82]. There were 36 proteins that were up-regulated while 22 proteins were found to be down-regulated in the serum-purified exosomes (SPEs) of patients with CRC by bioinformatics analysis. They revealed that the differently expressed proteins of SPEs from patients with CRC are vital for tumor invasiveness and play putative roles in pre-metastatic niche establishment, but exert minimal influence on putative alterations in tumor survival or proliferation [82]. Accordingly, this research might give impetus to clarify the patho-physiological functions of exosomes and the development of CRC diagnostics and therapeutics. Another study revealed that the tumor suppressor gene *TP53* changes the tumor microenvironment and promotes canceration via the endosomal sorting complex required for transport (ESCRT)-dependent exosome secretory machinery [83]. A comprehensive proteomic analysis identified that the expression of hepatocyte growth factor-regulated tyrosine kinase substrate (HGS) increases concomitantly with CRC tumorigenesis and is an independent poor prognostic factor, which may present itself as a novel biomarker and target for therapeutic interventions in CRC [83].

The delivery of exosomes to recipient cells could induce cell migration, inflammation, immune responses, angiogenesis, invasion, pre-metastatic niche formation, and metastasis [51–53, 84, 85]. Previous studies have shown that exosomes from mutant *KRAS* CRC cells can be transferred to wild-type cells to induce cell growth and migration [86, 87]. To test whether exosomal RNAs change the gene expression of recipient cells and whether mutant *KRAS* regulates the composition of the secreted miRNAs, researchers compared small RNAs of cells and matched exosomes from isogenic CRC cell lines differing only in the *KRAS* status [84]. Results showed that the *KRAS* status prominently affects the miRNA profile in cells and their corresponding exosomes, and cellular trafficking of miRNAs is sensitive to neutral sphingomyelinase (nSMase) inhibition in mutant *KRAS* cells as well as that the transfer of miRNAs between cells can functionally alter gene expression in recipient cells [84]. Similarly, researchers found that circular RNAs (circRNAs) were significantly down-regulated in DLD-1 and DKO-1 and HCT116 cell lines containing *KRAS* mutations, which indicated a widespread effect of mutant *KRAS* on circRNA abundance [88]. CircRNAs were more abundant in exosomes than in cells, which suggested that circRNAs may be promising biomarkers of CRC. Besides, CRC exosomes can induce morphological and functional changes in colonic mesenchymal stromal cells (MSCs), which may favor tumor growth and progression [89]. Exosomes can interact with target cells through specific receptor-ligand binding. Studies have shown that microvesicles from CRC released Fas ligand and tumor necrosis factor (TNF) to induce T-cell apoptosis, which can serve as a prognostic factor and can be targeted for novel antitumor therapies with regard to CRC [90].

As miRNAs may find applications in molecular therapies for the treatment of CRC [91], exosomes can be used as vectors for cancer intervention through gene or drug delivery. Dai et al. conducted a phase I clinical trial of the ascites-derived exosomes (Aex) in combination with the granulocyte-macrophage colony-stimulating factor (GM-CSF) in the immunotherapy of patients with CRC [92]. This treatment strategy could induce a beneficial, tumor-specific antitumor cytotoxic T lymphocyte (CTL) response, which may be an alternative choice in immunotherapy of advanced CRC [92]. Curiously, Zhao et al. provided evidence that cancer-associated fibroblast derived exosomes (CDEs) contain complete metabolites, including amino acids, lipids, and TCA-cycle intermediates. Cancer cells utilize central carbon metabolism of CDEs to promote tumor growth under nutrient deprivation or nutrient-stressed conditions, which may develop as a novel therapeutic concept for CRC [93]. Moreover, inhibiting the release of tumor exosomes or blocking the tumor exosome functions may also be a possible option to interfere with cancer. ExoDx^™^ Lung (*ALK*) from Exosome Diagnostics Company has been approved by the FDA in the United States and has become the world's first noninvasive test using exosomal RNA-based liquid biopsy. As one of the promising diagnostic and therapeutic tools for CRC in future, research on the mechanism of exosomes will lay the foundation for a better clinical application.

## CONCLUSIONS AND PERSPECTIVES

Tumorigenesis is the result of multiple factors implicated in the destruction of balance between oncogenes and anti-oncogenes. With approaches such as that of liquid biopsy, which are rapid, convenient, and minimally invasive, cancers can be detected from body fluid samples repeatedly, bypassing the need of tumor tissue biopsy while allowing clinicians to monitor the response to therapies and recurrence in real-time.

Although the early detection strategies and the guidance for decision-making during therapies based on liquid biopsy are promising, there have several drawbacks that need to be overcome before they can be applied clinically: 1) The surge in various detection technologies to identify CTCs, ctDNA, or exosomes, urgently calls for the implementation of standard guidelines, internal quality control (IQC) and external quality assessment (EQA). 2) Researchers need to find universal signatures from CTCs that cover any stage or type of cancer, thereby improving the sensitivity and specificity of the detection methods. 3) Large prospective clinical trials involving multicenter studies are needed to validate the clinical significance for detection and prognosis. 4) Complementary assessment of both CTCs and ctDNA will be more superior for the noninvasive diagnosis and prognosis monitoring of CRC. 5) There is an imposing need to explain the inconsistent results between liquid biopsy results and imaging examinations or tissue biopsies. Thus, liquid biopsies could help clinicians make better treatment-related decisions, provided that the above mentioned hurdles are overcome.

Fortunately, investigators have found that platelets might play a role in tumorigenesis and progression. Ratajczak and colleagues demonstrated that microparticles secreted by platelets induce angiogenesis and metastasis in both lung and breast cancer [16, 17]. Some researchers recently studied the total extracellular small RNA profiles from plasma, saliva, and urine of healthy subjects, which might contribute to the detection of health, disease, and injury [94]. Moreover, the occurrence of 8-Hydroxy-2’-deoxyguanosine (8-OHdG) and 5-Aminolevulinic Acid (5-ALA) or other such molecules in urine may potentially be set up as new screening or recurrence prognostic markers for CRC [95, 96, 97]. These could provide other promising directions for the detection of CRC.

In summary, owing to its significant benefits, liquid biopsy can be used as a feasible detection method for a variety of solid tumors and clinical diseases. Despite the existence of several challenges, we believe that the development of ideal detection methods is in progress. Our group has designed and conducted the first nationwide EQA of NGS-based targeted sequencing by laboratories in China in 2015 [98]. In addition, we are striving for the standardization of the detection of ctDNA by EQA. Undoubtedly, liquid biopsy will gain a foothold gradually and will be universally applied for clinical practice, after further optimization and improvement of technologies.

## Abbreviations

CRC: Colorectal cancer
CTCs: circulating tumor cells
ctDNA: circulating tumor DNA
FOBT: fecal occult blood test
CEA: carcinoembryonic antigen
CA19-9: carbohydrate antigen 19-9
MRI: Magnetic Resonance Imaging
CT: Computed Tomography
EMT: epithelial–mesenchymal transition
CTSCs: circulating tumor stem cells
FDA: Food and Drug Administration
EpCAM: epithelial cell adhesion molecule
RT-PCR: reverse transcription polymerase chain reaction
cfDNA: circulating free DNA
BEAMing: beads, emulsion, amplification and magnetics
dPCR: digital PCR
NGS: next generation sequencing
EBV: Epstein-Barr virus
NPC: nasopharyngeal carcinoma
mSEPT9: methylated *SEPT9* DNA
exoDNA: exosomal DNA
AS-PCR: allele-specific polymerase chain reaction
MBC: metastatic breast cancer
PFS: progression-free survival
OS: overall survival
mCRC: metastatic CRC
CKs: cytokeratins
NCCN: National Comprehensive Cancer Network
*EGFR*: epidermal growth factor receptor
*WIF1*: WNT inhibitory factor 1
NPY: neuropeptide Y
MetctDNA: methylated ctDNA
IonPGM: Ion Torrent PGM
miRNAs: microRNAs
CGH: comparative genomic hybridization
SPEs: serum-purified exosomes
ESCRT: endosomal sorting complex required for transport
HGS: hepatocyte growth factor-regulated tyrosine kinase substrate
nSMase: neutral sphingomyelinase
circRNAs: circular RNAs
MSCs: mesenchymal stromal cells
TNF: tumor necrosis factor
Aex: ascites-derived exosomes
GM-CSF: granulocyte-macrophage colony-stimulating factor
CTL: cytotoxic T lymphocyte
CDEs: cancer-associated fibroblast derived exosomes
IQC: internal quality control
EQA: external quality assessment
8-OHdG: 8-Hydroxy-2’-deoxyguanosine
5-ALA: 5-Aminolevulinic Acid
